# A chromosome-level genome assembly of the forestry pest *Coronaproctus castanopsis*

**DOI:** 10.1038/s41597-024-03016-6

**Published:** 2024-02-17

**Authors:** Yi-Xin Huang, Xiu-Shuang Zhu, Xiao-Nan Chen, Xin-Yi Zheng, Bao-Shan Su, Xiao-Yu Shi, Xu Wang, San-An Wu, Hao-Yuan Hu, Jian-Ping Yu, Yan-Zhou Zhang, Chao-Dong Zhu

**Affiliations:** 1 Key Laboratory of Zoological Systematics and Evolution, Institute of Zoology, Chinese Academy of Sciences, Beijing, 100101 China; 2 Qianjiangyuan National Park, Kaihua, Zhejiang 324300 China; 3 Collaborative Innovation Center of Recovery and Reconstruction of Degraded Ecosystem in Wanjiang Basin Co-founded by Anhui Province and Ministry of Education, School of Ecology and Environment, Anhui Normal University, Wuhu, Anhui 241000 China; 4 Beijing Forestry University, Beijing, 100083 China; 5 Anhui Provincial Key Laboratory of the Conservation and Exploitation of Biological Resources, College of Life Sciences, Anhui Normal University, Wuhu, Anhui 241000 China

**Keywords:** Entomology, Molecular biology, Systems biology

## Abstract

As an important forestry pest, *Coronaproctus castanopsis* (Monophlebidae) has caused serious damage to the globally valuable Gutianshan ecosystem, China. In this study, we assembled the first chromosome-level genome of the female specimen of *C. castanopsis* by merging BGI reads, HiFi long reads and Hi-C data. The assembled genome size is 700.81 Mb, with a scaffold N50 size of 273.84 Mb and a contig N50 size of 12.37 Mb. Hi-C scaffolding assigned 98.32% (689.03 Mb) of *C. Castanopsis* genome to three chromosomes. The BUSCO analysis (n = 1,367) showed a completeness of 91.2%, comprising 89.2% of single-copy BUSCOs and 2.0% of multicopy BUSCOs. The mapping ratio of BGI, second-generation RNA, third-generation RNA and HiFi reads are 97.84%, 96.15%, 97.96%, and 99.33%, respectively. We also identified 64.97% (455.3 Mb) repetitive elements, 1,373 non-coding RNAs and 10,542 protein-coding genes. This study assembled a high-quality genome of *C. castanopsis*, which accumulated valuable molecular data for scale insects.

## Background & Summary

Scale insects are highly adaptable to the surrounding environment and are widespread throughout the world, with more than 8520 species in 56 families (36 extant families and 20 extinct families) recorded to date. With the exception of a few resource species that can be applied to the chemical industry, such as *Ericerus pela*^1^, *Dactylopius coccus*^2^ and *Laccifer lacca*^3^, most scale insect are important agroforestry pests.

*Coronaproctus castanopsis* Li, Xu & Wu, 2023, was firstly discovered in Gutianshan National Nature Reserve, China^4^. Globally unique and undisturbed low-altitude subtropical evergreen broadleaf forests can be found in the Gutianshan Reserve. The field survey revealed that *C. castanopsis* are oligophagous and some of its main host plants are *Castanopsis eyrei*, *Castanopsis carlesii*, and *Castanopsis fargesii* (Fagaceae). These three species of trees are the primary constituents of the forest ecosystem in the reserve, and the scale insects mostly reside on the tree crowns, which are often difficult to observe. As a result, *C. castanopsis* has caused serious damage to the forest ecosystems of the Gutianshan Reserve.

The difficulty of high-quality scale insect genome assembly lies in its high degree of heterozygosity and a large number of repetitive sequences. There are only 13 coccoid genomes in the GenBank database, of which four species, mealybug - *Balanococcus diminutus, Phenacoccus solenopsis*, *Planococcus citri* and giant mealybug - *Icerya purchasi*, have been assembled into the chromosome-level genome. The limited availability of genomic data hindered our research on this group. Therefore, we constructed a chromosome-level *C. castanopsis* genome using a combination of BGI short reads, hifi long reads, and Hi-C data. We also annotated the genome for repetitive elements, protein-coding genes and non-coding RNAs, and performed phylogenetic and evolutionary analysis of the gene family. Our results contribute to the genome database of Coccomorpha and offer substantial support for a deeper understandingof *C. castanopsis* and future studies into scale insects.

## Methods

### Samples collection and sequencing

**Fig. 1:**
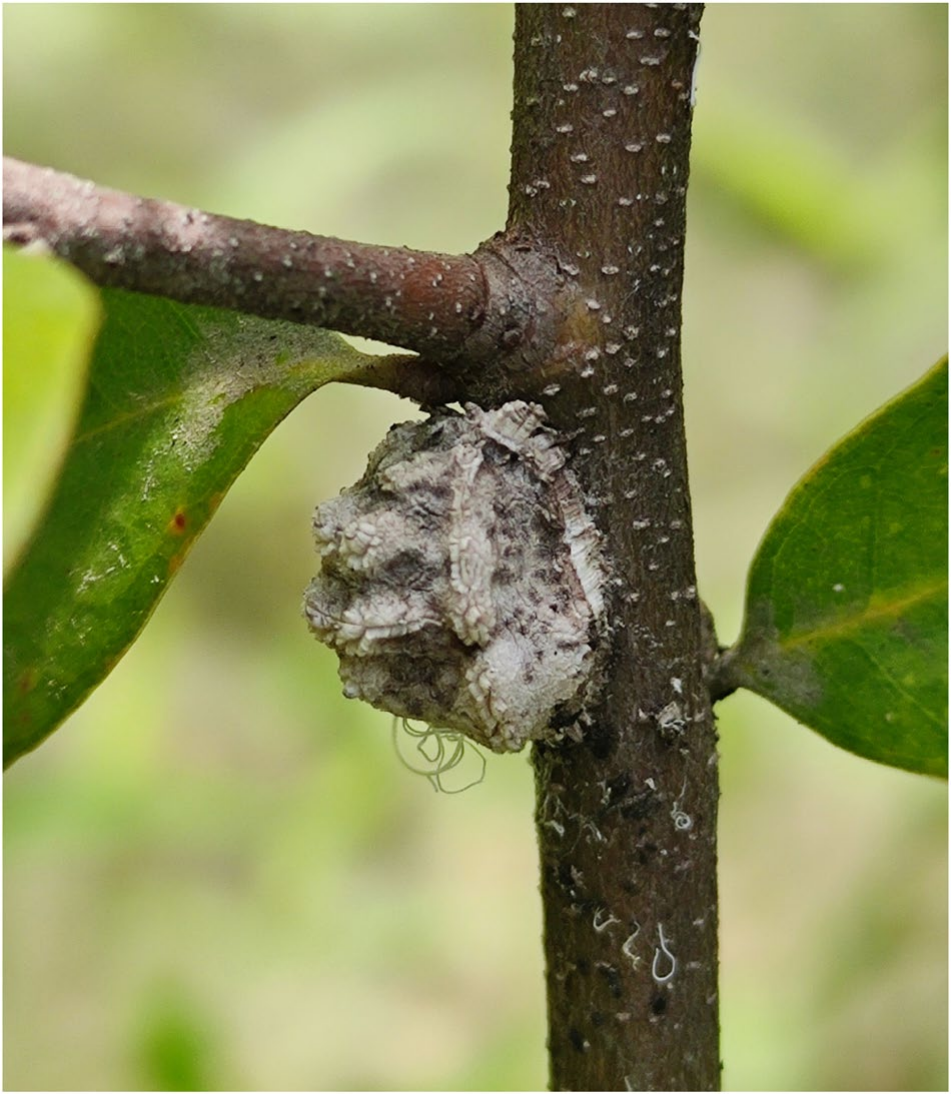
Ecological photo of a female adult *C. castanopsis* (photographed by Xiu-Shuang Zhu).

Adult female specimens of *C. castanopsis* (Fig. 1) were collected in May 2022 at Gutianshan National Nature Reserve (29.265° N, 118.101° E), Quzhou city, Zhejiang Province, China. Fresh samples were immediately placed in liquid nitrogen after collection and then stored at −80 °C for further use. To reduce contamination from gut microbes, we removed the metasoma of the samples and sent them to Berry Genomics Corporation (Beijing, China) for genome sequencing. The number of individuals used for genome survey, PacBio, Hi-C, and transcriptome sequencing was 10, 3, 5 and 5, respectively. Adult female specimens of *C. castanopsis* were used for transcriptome sequencing.

Genomic DNA, second-generation RNA and third-generation full-length RNA were extracted using the CTAB method^5^, the TRIzol TM Reagent Kit, and the RNA prep Pure Plant Plus Kit, respectively. The second-generation genome sequencing was completed on the Beijing Genomics Institute platform, and BGISEQ-500 library was constructed using the Agencourt AMPure XP-Medium Kit (insert size: 350 bp). PacBio HiFi sequencing was performed on the PacBio Sequel IIe platform, and the PacBio HiFi 15 K library was constructed using the SMRTbell® Express Template Prep Kit 2.0. Third-generation RNA sequencing (Oxford Nanopore Technologies (ONT) Oxford, UK) was performed on the Oxford Nanopore PromethION platform, and the ONT PromethION library was constructed using the SQK-PCS109 and SQKPBK004 kit. Both second-generation RNA (RNA-sr) and Hi-C library were performed on the Illumina NovaSeq. 6000 platform with 150-bp paired-end reads. We totally sequenced 183.99 Gb clean reads, including 36.75 Gb (53x) PacBio reads (N50 16.18 kb), 67.19 Gb (156x) BGI reads, 58.78 Gb (84x) Hi-C reads, 7.87 Gb second-generation RNA reads, and 13.40 Gb ONT RNA reads (Table 1).

**Table 1:** Sequencing data statistics for genome assembly.

Genomic libraries	Clean data (Gb)	Mean length (bp)	N50 (kb)	Sequencing coverage (X)
BGI	67.19	150	—	155.64
HiFi	36.75	16,184.60	16.18	52.50
Hi-C	58.78	150	—	83.97
RNA-sr	7.87	150	—	—
RNA-ONT	13.40	926.87	1.13	—

### Genome assembly

**Fig. 2:**
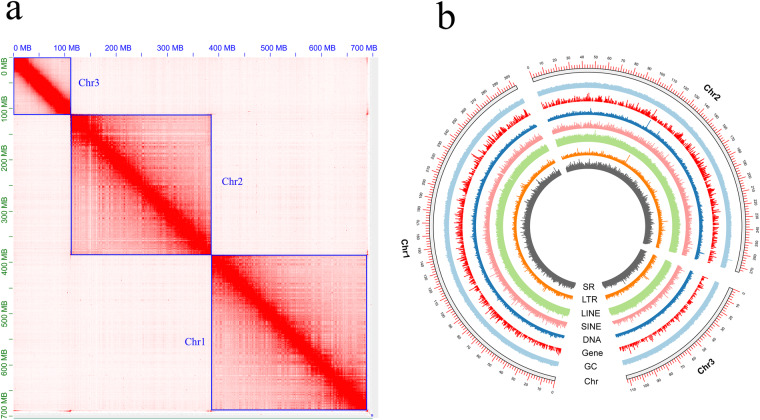
Genomic heatmap and features. (**a**) genome-scale chromosome heatmap of *C. castanopsis*, with individual chromosome outlined in blue. (**b**) circos plot with a window size of 100 Kbp. Each circle from inside to outside represents simple repeats, LTR, LINE, SINE, DNA, gene density, GC content and chromosome length.

High-quality HiFi reads (Q20 base quality) were generated by pbccs v6.4.0. Hifiasm v0.16.1^6^ was used for the first round of assembly with a parameter setting of “ -l 2”. The Hifiasm assembly only retained contig sequences with a sequencing depth of more than 10X to avoid possible errors or contamination. Minimap2 v2.24^7^ was used to paste the second-generation data back to the Hifiasm assembly, and SAMtools v1.10^8^ was used to convert the data format sam to bam. We also used NextPolish v1.4.0^9^ to perform short-read and long-read polishing to improve assembly accuracy. The Hi-C data and the 3D-DNA v180922^10^ process were used for chromosome mounting and assembly of contigs. After using Juicer v1.6.2^11^ to perform quality control on Hi-C data, we then performed two rounds of splicing using the default parameters of 3D-DNA v180922. Manual error correction was performed using Juicebox v1.11.08, and the sequencing depth of each pseudochromosome was evaluated by bamtocov v.2.7.0^12^. Genomic integrity was assessed by BUSCO v5.2.2^13^ based on the insecta_odb10 database (n = 1,367). Next, we used the postback tool Minimap2 to test the utilization of the original data and the integrity of the assembly, and the postback rate was counted by SAMtools v1.10. After polishing and correction, the final assembled genome size of *C. castanopsis* was 700.81 Mb, including 53 scaffolds and 161 contigs, with the scaffold/contig N50 size of 273.84/12.37 Mb and a GC content of 31.58% (Fig. 2a,b). In addition, Hi-C scaffolding assigned 98.32% (689.03 Mb) of *C. Castanopsis* genome to three pseudo-chromosomes (Fig. 2a). The BGI, second-generation RNA, third-generation RNA, and HiFi data reply rates were 97.84%, 96.15%, 97.96%, and 99.33%, respectively. The BUSCO analysis (n = 1,367) showed a completeness of 91.2%, comprising 89.2% of single-copy BUSCOs and 2.0% of multicopy BUSCOs. The above indicators showed that the assembly has reached a high level in terms of both continuity and integrity. We note that the values in the article may differ slightly in the final version of this assembly, where ~0.01% of the bases were removed or masked by the NCBI contamination screening program. In general, the genome of *C. castanopsis* has been assembled to a high degree of completeness.

### Genome annotation

Using RepeatMasker v4.1.2p1 (http://www.repeatmasker.org), we identified the repetitive regions of the genome against the final repetitive sequence reference database. The final repetitive sequence reference database included de novo repeat library, Dfam 3.5^14^ and RepBase-20181026^15^. The de novo repeat library was constructed using RepeatModeler v2.0.3^16^ and the ‘-LTRStruct’ search process. The results showed that the *C. castanopsis* genome contains about 64.97% (455.3 Mb) of repetitive elements, including LINEs (39.60%), unclassifed elements (13.15%), DNA transposons (6.33%), LTR elements (1.39%), simple repeats (2.68%), and other elements (Table S1).

To predict and identify the protein-coding gene structure, we used MAKER v3.01.03^17^ to integrate three types of strategies (ab initio prediction, transcript sequence alignment and homologous proteins comparison). Input files for MAKER ab initio were obtained by using BRAKER v2.1.6^18^ and GeMoMa v1.8^19^ and integrating both transcriptomic and protein evidence. Transcriptome alignments were generated by using HISAT2 v2.2.0^20^. Two predictors, Augustus v3.3.4^21^ and GeneMark-ES/ET/EP 4.68_3.60_lic^22^, were automatically trained by BRAKER based on reference proteins mined from the OrthoDB10 v1 database^23^ and transcriptome data. Using information on protein homology and intron location, GeMoMa was used to predict genes with the parameter of “GeMoMa.c = 0.4GeMoMa.p = 10” and the protein sequences of five related species (*Tribolium castaneum*, *Coccinella septempunctata*, *Apis mellifera*, *Chrysoperla carnea* and *Drosophila melanogaster*). Reference assembly (–mix) based on second and third-generation transcriptomes was performed using StringTie v2.1.6^24^, and RNA sequences alignments were generated by HISAT2. Besides, predictions were made in GeMoMa via homology comparison with the protein sequences of the five species above. In total, the MAKER process identified 10,542 protein-coding genes with an average gene length of 19,827.3 bp. The average number of exons (mean length: 294.3 bp), introns (mean length: 2629.6 bp) and CDS (mean length: 208 bp) in each gene was 7.8, 6.8 and 7.5, respectively. The predicted protein gene sequences assessed for BUSCO completeness were 91.2% (n: 1367), including 78.8% single-copy, 12.4% duplicated, 0.7% fragmented and 8.1% missing BUSCOs.

Using the high-sensitivity mode (–very-sensitive -e 1e-5) in Diamond v2.0.11.149^25^, we searched the UniProtKB database for protein-coding gene function annotation. In addition, in order to annotate Gene Ontology (GO) and (KEGG, Reactome) pathways and identify protein domains, we searched Pfam^26^, SMART^27^,Superfamily^28^ and CDD^29^ databases using InterProScan 5.53–87.0^30^, and we also searched the eggNOG v5.0^31^ database using eggNOG-mapper v2.1.5^32^. Finally, Genes with 8363 GO terms, 4217 KEGG pathways, 2474 Enzyme Codes, 7982 Reactome pathways, and 9323 COG categories were identified by combining the eggNOG and InterProScan annotation results (Table S2).

The annotations of rRNA, snRNA and miRNA were compared with the Rfam database using Infernal v1.1.4^33^. Prediction of tRNA sequences was performed using tRNAscan-SE v2.0.9^34^, with low confidence tRNAs filtered by the ‘EukHighConfidenceFilter’ script. We totally identifed 1373 ncRNAs in the genome of *C. castanopsis*, including 265 ribosomal RNAs, 52 microRNAs, 22 small RNAs, 40 long non-coding RNA, 515 small nuclear RNAs, 153 transfer RNAs, and 326 other ncRNAs (Table S3).

## Data Records

The raw sequencing data and genome assembly of *Coronaproctus castanopsis* have been submitted to the National Center for Biotechnology Information (NCBI) and the China National GeneBank DataBase (CNGBdb). The Hi-C, PacBio, RNA-ONT, RNA-sr and BGI data are accessible via accession numbers SRR26067557-SRR26067561^35–39^. The BGI, RNA-sr, RNA-ONT, PacBio and Hi-C data are accessible via accession numbers CNX0846626-CNX0846630^40–44^. The assembled genome is accessible via accession number GCA_032883995.1^45^.

## Technical Validation

The assessment of the quality of the genome assembly has been a two-step process. Initially, we assessed the completeness of the assembly using BUSCO v5.2.2 based on the insecta_odb10 database (n = 1,367). The final genome assembly displayed a BUSCO completeness of 91.2%, comprising of 1219 (89.2%) single-copy BUSCOs, 27 (2.0%) duplicated BUSCOs, 33 (2.4%) fragmented BUSCOs, and 87 (6.4%) missing BUSCOs. We then calculated the mapping rate to measure the accuracy of the assembly. The BGI, second-generation RNA, third-generation RNA, and Hifi data reply rates were 97.84%, 96.15%, 97.96%, and 99.33%, respectively. Overall, these assessments reflect the high quality of the genomic assembly.

### Supplementary information

Supplementary Information

## Data Availability

No specific script was used in this work. All commands and pipelines used in data processing were executed according to the manual and protocols of the corresponding bioinformatic softwares.
