# Supplementary material for: A chromosome-level genome assembly of the forestry pest Coronaproctus castanopsis
Source: Sci Data. 2024 Feb 17;11:218. doi: 10.1038/s41597-024-03016-6 (PMC10874433; doi:10.1038/s41597-024-03016-6)
Supplement: Supplementary file 1 — Supplementary Information [file 41597_2024_3016_MOESM1_ESM.pdf]

CONTENTS

Table S1 1
Table S1 6
Table S3 8

Table S1 Repetitive elements in the genome of *Coronaproctus castanopsis*

Class	Copies	Length (bp)	Percentage of the genome (bp)
**ARTEFACT**	353	214510	0.03%
**DNA**	6910	371477	0.05%
Academ	21	6543	0.00%
Academ-1	585	67270	0.01%
Academ-2	8	554	0.00%
Academ-H	3	188	0.00%
CMC	1	59	0.00%
CMC-Chapaev	2992	121445	0.02%
CMC-Chapaev-3	524	91948	0.01%
CMC-EnSpm	39803	2348757	0.34%
CMC-Mirage	2	113	0.00%
CMC-Transib	2032	148828	0.02%
Crypton	896	35431	0.01%
Crypton-A	49	1950	0.00%
Crypton-F	4	145	0.00%
Crypton-H	1609	59552	0.01%
Crypton-I	1	42	0.00%
Crypton-S	17	893	0.00%
Crypton-V	3035	103970	0.01%
Dada	2874	96836	0.01%
Ginger-1	2809	123082	0.02%
Ginger-2	14	680	0.00%
IS3EU	3061	152919	0.02%
Kolobok	22	988	0.00%
Kolobok-E	15	773	0.00%
Kolobok-H	10	554	0.00%
Kolobok-Hydra	173	7950	0.00%
Kolobok-T2	5516	232116	0.03%
MULE-F	2	91	0.00%
MULE-MuDR	14603	1370692	0.20%
MULE-NOF	577	103127	0.01%
Maverick	10398	5305076	0.76%
Maverick-Mavirus	1	66	0.00%
Merlin	2412	524100	0.07%
Novosib	165	6879	0.00%
P	7750	2180358	0.31%
P-Fungi	5	260	0.00%

PIF-HarbS	1	43	0.00%
PIF-Harbinger	6387	954480	0.14%
PIF-ISL2EU	235	9227	0.00%
PIF-Spy	460	17597	0.00%
PiggyBac	12966	4840970	0.69%
PiggyBac-A	5	277	0.00%
PiggyBac-X	9	318	0.00%
Sola-1	424	36568	0.01%
Sola-2	631	23149	0.00%
Sola-3	253	11603	0.00%
TcMar	433	15750	0.00%
TcMar-Ant1	11	599	0.00%
TcMar-Cweed	2	87	0.00%
TcMar-Fot1	3244	248442	0.04%
TcMar-IS885	6	213	0.00%
TcMar-ISRm11	508	20868	0.00%
TcMar-Mariner	1999	700113	0.10%
TcMar-Pogo	4837	2474178	0.35%
TcMar-Sagan	5	197	0.00%
TcMar-Stowaway	163	5503	0.00%
TcMar-Tc1	31736	8269678	1.18%
TcMar-Tc2	975	507591	0.07%
TcMar-Tc4	191	8157	0.00%
TcMar-Tigger	5326	1639926	0.23%
TcMar-m44	25	912	0.00%
Zator	23	1191	0.00%
Zisupton	12413	571153	0.08%
hAT	2825	352931	0.05%
hAT-Ac	9338	2006037	0.29%
hAT-Blackjack	1598	251345	0.04%
hAT-Charlie	6715	1516069	0.22%
hAT-Pegasus	95	4503	0.00%
hAT-Restless	3	116	0.00%
hAT-Tag1	790	31593	0.00%
hAT-Tip100	19191	6325004	0.90%
hAT-hAT1	1	34	0.00%
hAT-hAT19	769	185274	0.03%
hAT-hAT5	45	2181	0.00%
hAT-hAT6	5	214	0.00%
hAT-hATm	940	33961	0.00%

hAT-hATw	194	7681	0.00%
hAT-hATx	27	1436	0.00%
hAT-hobo	50	3037	0.00%
**LINE**	13409	5567667	0.79%
CR1	11692	4460987	0.64%
CR1-Zenon	365	140029	0.02%
CRE	223	13037	0.00%
CRE-Ambal	108	5290	0.00%
CRE-Odin	22	1133	0.00%
Deceiver	7	350	0.00%
Dong-R4	1490	501518	0.07%
Dualen	8	303	0.00%
I	119483	56089115	8.00%
I-Jockey	183197	90138102	12.86%
L1	7389	311973	0.04%
L1-DRE	202	9377	0.00%
L1-Tx1	1743	320092	0.05%
L1-Zorro	1	69	0.00%
L2	36028	9295572	1.33%
Penelope	41297	7711477	1.10%
Proto1	119	5708	0.00%
Proto2	23	1093	0.00%
R1	39933	16826106	2.40%
R1-LOA	998	482565	0.07%
R2	2690	161567	0.02%
R2-Hero	87	3628	0.00%
R2-NeSL	710	47059	0.01%
RTE	6897	2394633	0.34%
RTE-BovB	181157	69660636	9.94%
RTE-ORTE	14	543	0.00%
RTE-RTE	36509	12988139	1.85%
RTE-X	1400	254449	0.04%
Rex-Babar	163	6188	0.00%
Tad1	1542	203417	0.03%
**LTR**	425	14752	0.00%
Caulimovirus	114	5997	0.00%
Copia	4847	2133781	0.30%
DIRS	298	12374	0.00%
DIRS-Q	4	181	0.00%
ERV-Foamy	3	193	0.00%
ERV-Lenti	3	170	0.00%
ERV1	3696	144918	0.02%
ERV4	280	9909	0.00%
ERVK	1546	63771	0.01%

ERVL	1594	801079	0.11%
ERVL-MaLR	4	198	0.00%
Gypsy	23701	5336012	0.76%
Ngaro	772	33073	0.00%
Pao	2911	1305112	0.19%
Viper	4	170	0.00%
**Other**	--	--	--
DNA_virus	414	230506	0.03%
**RC**	--	--	--
Helitron	25191	5846482	0.83%
Helitron-2	64	1840	0.00%
**Retroposon**	11	695	0.00%
L1-dep	47	1732	0.00%
L1-derived	1	75	0.00%
L2-derived	4	227	0.00%
R4-derived	1	42	0.00%
RTE-derived	71	2284	0.00%
sno	72	2456	0.00%
**SINE**	3	245	0.00%
5S	2103	202365	0.03%
5S-Core-RTE	4240	655313	0.09%
5S-Deu-L2	1	23	0.00%
5S-RTE	1	54	0.00%
5S-Sauria-RTE	2	98	0.00%
7SL	3	735	0.00%
Alu	104	3852	0.00%
B2	29	947	0.00%
B4	77	2434	0.00%
Ceph	1	53	0.00%
Core	1	58	0.00%
ID	97	3223	0.00%
MIR	12	567	0.00%
U	3696	474416	0.07%
U-L1	132	8621	0.00%
tRNA	617	23086	0.00%
tRNA-5S	6	100	0.00%
tRNA-7SL	3	41	0.00%
tRNA-Core	226	6980	0.00%
tRNA-Core-RTE	1	9	0.00%
tRNA-Deu-I	1	47	0.00%
tRNA-Deu-L2	1	64	0.00%
	6	214	0.00%

tRNA-Deu-RTE			
tRNA-I	6	134	0.00%
tRNA-L2	10	272	0.00%
tRNA-Meta	57	1543	0.00%
tRNA-R2	1	15	0.00%
tRNA-RTE	20204	3069616	0.44%
Segmental	1	48	0.00%
**Unknown**	512968	92140833	13.15%
centromeric	2	90	0.00%
Total_interspersed	1534702	434906375	62.06%
**Low_complexity**	28683	1354028	0.19%
**Satellite**	2098	95896	0.01%
acro	12	488	0.00%
macro	365	9655	0.00%
subtelo	1	42	0.00%
**Simple_repeat**	396097	18804677	2.68%
**rRNA**	156	100636	0.01%
**snRNA**	369	18174	0.00%
**tRNA**	196	11141	0.00%
**Total**	1962679	455301112	64.97%

Table S2 Annotation of protein-coding genes in the genome of *Coronaproctus castanopsis*

Class	
**Structure annotation**	
Number of protein-coding genes	10,542
Number of predicted protein sequences	12,331
Mean protein length (aa)	519.4
Mean gene length (bp)	19,827.30
Gene ratio	29.83%
Number of exons per gene	7.8
Mean exon length (bp)	294.3
Exon ratio	3.48%
Number of CDSs per gene	7.5
Mean CDS length (bp)	208
CDS ratio	2.37%
Number of introns per gene	6.8
Mean intron length (bp)	2629.6
Intron ratio	26.35% (26.99%)
**Function annotation**	
Number of genes matching Uniprot records	9667
Number of genes labelled as "Uncharacterized protein"	595
Number of genes labelled as "unknown function"	954
Number of genes with InterProScan annotations	9138
Number of genes with GO items from InterProScan annotations	5886
Number of genes with KEGG pathway items from InterProScan annotations	0
Number of genes with MetaCyc items from InterProScan annotations	6326
Number of genes with Reactome items from InterProScan annotations	7982
Number of genes with eggNOG annotations	9679
Number of genes with GO items from eggNOG annotations	7382
Number of genes with Enzyme Codes (EC) from eggNOG annotations	2474
Number of genes with KEGG ko terms from eggNOG annotations	6759
Number of genes with KEGG pathway terms from eggNOG annotations	4217
Number of genes with COG Functional Categories from eggNOG annotations	9323

Number of genes with GO items (combining InterProScan and eggNOG results)	8363
Number of genes with KEGG pathways items (combining InterProScan and eggNOG results)	4217

Table S3 Noncoding RNAs in the genome of *Coronaproctus castanopsis*

Class	Copies	Description
**rRNA**		
5_8S_rRNA	1	rRNA
5S_rRNA	225	rRNA
LSU_rRNA_bacteria	4	rRNA
LSU_rRNA_eukarya	15	rRNA
SSU_rRNA_bacteria	6	rRNA
SSU_rRNA_eukarya	14	rRNA
	265	
**miRNA**		
bantam	1	miRNA
mir-1	1	miRNA
mir-1000	1	miRNA
mir-1175	1	miRNA
mir-190	1	miRNA
mir-1923	28	miRNA
mir-219	1	miRNA
mir-242	2	miRNA
mir-263	1	miRNA
mir-276	1	miRNA
mir-277	2	miRNA
mir-317	1	miRNA
mir-449	1	miRNA
mir-46	1	miRNA
MIR480	1	miRNA
mir-643	2	miRNA
mir-67	1	miRNA
mir-7	1	miRNA
mir-71	1	miRNA
mir-9	1	miRNA
mir-929	1	miRNA
mir-iab-4	1	miRNA
	52	
**sRNA**		
CyaR_RyeE	2	sRNA
GlmZ_SraJ	1	sRNA
isrK	11	sRNA
MicA	1	sRNA
P26	1	RF00630
Ricks_sRNA10	2	sRNA
SraG	1	sRNA
t44	1	sRNA
tpke11	1	sRNA

WsnRNA59	1	sRNA
	22	
**snRNA**		
SNORD18	3	snRNA; snoRNA; CD-box
SNORD31	3	snRNA; snoRNA; CD-box
SNORD33	3	snRNA; snoRNA; CD-box
SNORD36	1	snRNA; snoRNA; CD-box
snosnR60_Z15	5	snRNA; snoRNA; CD-box
U1	5	snRNA; splicing
U11	1	snRNA; splicing
U12	1	snRNA; splicing
U2	366	snRNA; splicing
U3	17	snRNA; snoRNA; CD-box
U4	22	snRNA; splicing
U4atac	2	snRNA; splicing
U5	17	snRNA; splicing
U6	63	snRNA; splicing
U6atac	6	snRNA; splicing
	515	
**lncRNA**		
ribozyme		
RNaseP_bact_a	5	ribozyme
RNaseP_nuc	1	ribozyme
**riboswitch**		
FMN	1	riboswitch
M-box	1	riboswitch
MOCO_RNA_motif	2	riboswitch
yybP-ykoY	1	riboswitch
**leader**		
L25-Gammaproteobacteria	1	leader
L31-Gammaproteobacteria	1	leader
Phe_leader	1	leader
rimP	1	leader
RpsF_leader	1	leader
S15	1	leader
S16-Flavobacteria	1	leader
**antisense**		
AsdA	1	antisense
C4	16	antisense
HPnc0260	2	antisense

Ysr276	1	antisense
**thermoregulator**		
CpoB_ybgF_thermometer	1	thermoregulator;
cspA	1	thermoregulator
**tRNA**		
tRNA-Ala	16	tRNA
tRNA-Arg	19	tRNA
tRNA-Asn	10	tRNA
tRNA-Asp	5	tRNA
tRNA-Cys	3	tRNA
tRNA-Gln	6	tRNA
tRNA-Glu	6	tRNA
tRNA-Gly	7	tRNA
tRNA-His	5	tRNA
tRNA-Ile	4	tRNA
tRNA-iMet	3	tRNA
tRNA-Leu	9	tRNA
tRNA-Lys	7	tRNA
tRNA-Met	4	tRNA
tRNA-Phe	3	tRNA
tRNA-Pro	6	tRNA
tRNA-Ser	12	tRNA
tRNA-Thr	12	tRNA
tRNA-Trp	3	tRNA
tRNA-Tyr	5	tRNA
tRNA-Val	8	tRNA
**Others**		
6S	1	6S / SsrS RNA
Alpha_RBS	1	Alpha operon ribosome binding site
Bacteria_small_SRP	1	Bacterial small signal recognition particle RNA
group-II-D1D4-1	34	Group II catalytic intron D1-D4-1
group-II-D1D4-3	33	Group II catalytic intron D1-D4-3
group-II-D1D4-7	3	Group II catalytic intron D1-D4-7
Histone3	92	Histone 3' UTR stem-loop
Intron_gpII	3	Group II catalytic intron
K_chan_RES	5	Potassium channel RNA editing signal
Metazoa_SRP	2	Metazoan signal recognition particle RNA
R2_retro_el	145	R2 RNA element
RtT	1	RtT RNA
tmRNA	5	transfer-messenger RNA
**Total**	**1373**	
